# Assessing the Nationwide COVID-19 Risk in Mexico through the Lens of Comorbidity by an XGBoost-Based Logistic Regression Model

**DOI:** 10.3390/ijerph191911992

**Published:** 2022-09-22

**Authors:** Sonia Venancio-Guzmán, Alejandro Ivan Aguirre-Salado, Carlos Soubervielle-Montalvo, José del Carmen Jiménez-Hernández

**Affiliations:** 1 Institute of Physics and Mathematics, Universidad Tecnológica de la Mixteca, Huajuapan de León C.P. 69000, Mexico; 2 Faculty of Engineering, Universidad Autónoma de San Luis Potosí, San Luis Potosí C.P. 78280, Mexico

**Keywords:** coronavirus, comorbidity, spatial analysis, logistic regression, ROC curve

## Abstract

The outbreak of the new COVID-19 disease is a serious health problem that has affected a large part of the world population, especially older adults and people who suffer from a previous comorbidity. In this work, we proposed a classifier model that allows for deciding whether or not a patient might suffer from the COVID-19 disease, considering spatio-temporal variables, physical characteristics of the patients and the presence of previous diseases. We used XGBoost to maximize the likelihood function of the multivariate logistic regression model. The estimated and observed values of percentage occurrence of cases were very similar, and indicated that the proposed model was suitable to predict new cases (AUC = 0.75). The main results revealed that patients without comorbidities are less likely to be COVID-19 positive, unlike people with diabetes, obesity and pneumonia. The distribution function by age group showed that, during the first and second wave of COVID-19, young people aged ≤20 were the least affected by the pandemic, while the most affected were people between 20 and 40 years, followed by adults older than 40 years. In the case of the third and fourth wave, there was an increased risk for young individuals (under 20 years), while older adults over 40 years decreased their chances of infection. Estimates of positive COVID cases with both the XGBoost-LR model and the multivariate logistic regression model were used to create maps to visualize the spatial distribution of positive cases across the country. Spatial analysis was carried out to determine, through the data, the main geographical areas where a greater number of positive cases occurred. The results showed that the areas most affected by COVID-19 were in the central and northern regions of Mexico.

## 1. Introduction

The COVID-19 pandemic has affected many countries worldwide. Among these, the United States, India, Brazil, the United Kingdom and Russia lead the list of countries with the highest number of infected [1]. The outbreak of the SARS-CoV-2 virus was declared a pandemic in March 2020, due to its rapid dissemination and its negative effects across various countries [2]. After 18 months, the United States has reported 82,613,620 confirmed cases and 999,842 deaths; India has reported 43,125,370 confirmed cases and 524,260 deaths; Brazil has reported 30,701,900 confirmed cases and 665,216 deaths, among others. In the case of Mexico (5,752,441 confirmed cases and 324,617 deaths), it is in 15th and 4th place regarding the countries with the larger number of positive cases and deaths, respectively [1].

Coronaviruses are a group of viruses that cause respiratory illnesses [3]. The most common symptoms produced by the virus include fever, dry cough, tiredness and shortness of breath, though, in more severe cases, it causes pneumonia or severe acute respiratory syndrome (SARS) that may lead to death. In other cases, some people infected by the virus do not develop any symptom, but they may still infect the rest of the population. According to the World Health Organization (WHO), the coronavirus can be transmitted from an infected person to others through droplets (also called aerosols) that are expelled when coughing, sneezing or speaking, when shaking a sick person’s hand, or by touching objects or surfaces contaminated by the virus [4].

The vulnerability of a person to COVID-19 is determined by various factors; among these, the educational level, age, gender, social conditions, sociodemographic conditions, housing conditions, and even psychological and emotional factors. In their recent study, Ref. [5] analyzed the effects of living conditions on morbidity and mortality in the state of Oaxaca. In this study, it was determined that the health of the population was seriously affected by poor living conditions, and the lack of services such as electricity, gas, water, and health educational services. Therefore, several factors have contributed to the exacerbation of the coronavirus disease. Ref. [6] tested the hypothesis that social vulnerability in Mexico contributes considerably to the probability of hospitalization of people sick from COVID-19. To prove this, a cross-sectional study was carried out with public data from the General Directorate of Epidemiology of the Ministry of Health of Mexico using 5-month period data from individuals with coronavirus. The results show that patients with diabetes have 38.4% probability of being hospitalized, and for those patients whose suffer from some other comorbidity, the risk increases to 42.9% (42.2–43.7%).

Several studies have carried out for the evaluation of comorbidities associated with severe and fatal cases of COVID-19. Ref. [7] reviewed 33 systematic studies and 22 meta-analysis, concluding that, of the total cases, 40% had comorbidities, while fatal cases had about 74% had comorbidities. Hypertension, diabetes, and respiratory diseases were the most frequent comorbidities in severe and fatal cases. Ref. [8] founded that about 66.6% of the deaths caused by from COVID-19 were to men, with a mean age of 69.9 years. In addition, a high incidence of hypertension, diabetes, low platelet levels, and chronic cerebrovascular and cardiovascular diseases were observed among the deceased. Ref. [2] carried out a descriptive analysis of COVID-19 cases in Mexico with data obtained through the official website of the Ministry of Health in Mexico. The study included epidemiological, demographic, and clinical characteristics of the patients who were confirmed positive by a real-time RT-PCR test. The results determined that most of the positive cases of COVID-19 occurred in Mexico City, the average age of the patients confirmed with the disease was 46 years old, most of these cases occurred in people between 30 to 59 years, and 58% of all positive cases occurred in men. Regarding deceased patients, it was determined that they had one or more comorbidities, mainly hypertension, diabetes and obesity. Similarly, Ref. [9] determined that the most influential factor in men and women was obesity, followed by diabetes and hypertension, as well as chronic kidney failure only in the case of women. These findings indicate that these comorbidities were associated with the severity of the disease and predispose to more serious complications of COVID-19.

Multivariate logistic regression has been widely used to identify variables associated with risks of coronavirus disease. Ref. [10] adjusted a multivariable logistic regression models with 450 patients from the Massachusetts General Hospital (MGH). They found that, among patients with diabetes, 42.1% was admitted to the intensive care unit, or ICU, 37.1% required mechanical ventilation, and 15.9% died; while, among patients without diabetes, 29.8% was admitted to ICU, 23.2% required mechanical ventilation, and 7.9% died. In their results, they also found that diabetes and obesity were associated with greater odds of ICU admission and mechanical ventilation. Ref. [11] conducted a study with 220 adult patients with confirmed and suspected COVID-19 using multivariate logistic regression and found that older age was an independent risk factor for mortality. In a study with 648 COVID-19-positive patients with a median age of 34 years, using multivariate logistic regression, Ref. [12] found that independent risk factors for critical outcomes among COVID-19 cases include old age, males, cardiac patients, chronic respiratory diseases, and the presence of two or more comorbidities; all had significant *p*-values < 0.05. In order of predicting COVID-19 severity, Ref. [13] conducted a retrospective study with 287 patients of which 36.6% were classified as severe cases and 63.4% as non-severe cases. They used 23 covariates on blood chemistry and obtained an accuracy of 85.2% and an AUC of 0.928. Ref. [14] analyzed the survival probability of patients with COVID-19 in Mexico considering their characteristics and comorbidities. They carried out logistic regression analyses and fitted nonparametric survival curves using the Kaplan–Meier estimator. A greater risk of death was associated with diabetes, obesity, hypertension, age and gender, and at a lower degree to, obstructive pulmonary disease, kidney disease and immunosuppressive diseases. Ref. [15] implemented two logistic regression models to investigate the rate of hospitalization and mortality against other variables. In the analysis, 10,544 records published by the Epidemiological Surveillance System of Respiratory Viral Diseases of the Ministry of Health of Mexico (Ministry of Health, SSA) were used. The results showed that the majority of the positive cases were men, being 54% times more likely to be hospitalized than women. People older than 50 years and with two or more simultaneous chronic diseases were prone to a higher risk of hospitalization and death.

Currently, there is a wide variety of statistical models and machine learning techniques that allow predictions to be made given a set of characteristics. Ensemble learning uses simple models to form a more precise and efficient algorithm. Boosting is an ensemble technique where simpler algorithms are used in a sequential way to take advantage of each original model in order to improve the precision of the subsequent model (Freund and Shapire, 1996). Some assembly models that are based on the Boosting principle include the AdaBoost [16], CatBoost [17], LightGBM [18] and Extreme Gradient Boosting (XGBoost). XGBoost is an algorithm developed as a research project at the University of Washington by [19] at the SIGKDD conference. This model was built by adding simpler models based on regression trees. The optimization algorithm consisted on the gradient descent on an objective function composed of the sum of individual loss functions of each observation. These loss functions measure the distance between an observation and its prediction based on the sum of its estimate in the previous iteration plus a new function that is added sequentially in each iteration. These characteristics allow the global estimation of the model to be scalable.

The XGBoost algorithm has proven its effectiveness and scalability over other methods in recent studies. For example, Ref. [20] implemented XGBoost to predict PM2.5 concentration per hour. The method was compared to several algorithms including random forest, multiple linear regression, decision tree, and support vector machines. Their results showed that this algorithm superpassed all of these methods. Ref. [21] designed a real driving task to extract data to model driving stress dynamics. They built a driver stress management model based on driving behavior, environment and road familiarity, and performed a cluster analysis with K-means to grouping observations of psychological and driver stress data. The XGBoost was used to monitor stress within each group. Performance comparisons revealed that the XGBoost model significantly transcended support vector machines, random forest and gradient boosting decision tree.

In accordance with the current health emergency around the world, it is necessary to build models that can predict the characteristics of people at higher risk of being infected with COVID-19. Therefore, in this research work, we build an XGBoost model to predict when the patient may or may not present the disease, considering risk factors such as having previous diseases. For this, the proposed methodology to address this problem is initially described. Subsequently, the description of the data is made, as well as the main results obtained from the descriptive statistics and the results predicted by the model. Finally, a brief discussion and conclusion is made about the main results obtained, as well as the scope and limitations of the work carried out.

## 2. Materials and Methods

The population of Mexico is approximately 126 million people, with a proportion of 51.2% and 48.8% of women and men (Population and Housing Census 2020, [22]), with a median of 29 years old. The country has 32 states, of which the state of Mexico has the largest number of inhabitants, while the state of Colima is the least populated. The data used to carry out this study were collected for the entire country.

### 2.1. Available Data

The data for this study were obtained from the official website of the General Directorate of Epidemiology of the Ministry of Health of Mexico https://www.gob.mx/salud/documentos/datos-abiertos-152127 (accessed on 10 May 2022). The records obtained from the database range from February 2020 to the end of April 2022, a total of 15,119,419 registered cases, and 40 explanatory variables. The set of variables used is shown in Table 1. The only available geographic data were the name of the municipality. To obtain the geographic coordinates of the municipalities, a shapefile was used. In order to merge the data, 7,347,705 were eliminated from the database, this due to the presence of missing values on the records and the presence of variables that represented individuals with duplicate characteristics, thus leaving a total of 7,771,714 individuals to be considered for analysis; of these, 3,119,851 (40.14%) was confirmed as positive cases and the rest as negative cases.

### 2.2. Logistic Regression

A generalized linear model is a function that relates a set of response variables with some distribution belonging to the exponential family and a set of independent variables that are obtained through a measurement or observation. This type of model allows the generalization of other existing models by varying the distribution of the response variable or the function that relates $E\left(Y_{i}\right)=\mu_{i}$ with the set of covariates [23]. This last function, commonly called the link function, is a monotonic and differentiable function, which satisfies:

(1) $$g\left(\mu_{i}\right)=X^{⊤}\beta ,$$

where *X* is an nxp matrix of explanatory covariates and $\beta \in R^{p}$ is a px1 vector of coefficients of the model. In the case where the response variable follow a Bernoulli distribution, the parameter $\mu_{i}=p_{i}$ represents the probability that a success will occur. The link function commonly used in this case is the *logit* function $g\left(\mu \right)=g\left(p\right)=\log\left(\frac{p}{1-p}\right).$ Consequently, the conditional probability of *Y* given *X* can be expressed as:

(2) $$p\left(Y=y|X\right)=\frac{e^{X^{⊤}\beta }}{1+e^{X^{⊤}\beta }}=\frac{e^{\beta_{0}+\beta_{1}x_{1}+\beta_{2}x_{2}+\ldots +\beta_{p}x_{p}}}{1+e^{\beta_{0}+\beta_{1}x_{1}+\beta_{2}x_{2}+\ldots +\beta_{p}x_{p}}},$$

which satisfies (1). This model is known as the logistic regression model.

### 2.3. XGBoost

In this research, we used an XGBoost-based logistic regression model for the classification problem, which may be denoted XGBoost-LR, where the explanatory variables are the main risk factors described in [7,8], and the response variable is the presence or absence of COVID-19 disease.

Let be a data set: $\left\{\left(x_{i},y_{i}\right):i=1,\ldots ,n,\;x_{i}\in R^{p},\;y_{i}\in R\right\}$, where $x_{i}$ is the vector of characteristics, and $y_{i}$ is the response variable. An assembly model based on regression trees makes use of *K* simpler models, to predict $y_{i}$ additively:

$${\hat{y}}_{i}=\sum_{k=1}^{K}f_{k}\left(x_{i}\right),\;f_{k}\in F,$$

${\hat{y}}_{i}$ is the prediction of $y_{i}$ and **F** is the class of functions of all possible regression trees;

(3) $$F=\left\{f\left(x\right)=w_{q(x)}|\;q:R^{p}\to I,\;w\in R^{T}\right\},$$

*T* is the number of leaf, I={1,2,…,T}, q(x) represents the index q(x)-th in vector *w*, $w_{q(x)}$ represents the q(x)-th component of *w*, $f_{k}$ represents an independent regression tree that corresponds to a tree structure *q* with leaf weights *w*.

Regression trees have a score on each of their leaves, $w_{i}$ denotes the score on the *i*-th leaf of a tree. The data used in the training of the model are grouped in the leaf nodes. Thus, in the prediction of an example, the decision rules generated by these trees are considered to classify it in the corresponding leaves and calculate the final prediction adding each of the scores in the leaves given by the vector *w* of each tree [19].

The regularized function to minimize, and to learn each $f_{i}$ is:

(4) $$\begin{array}{c} L=\sum_{i=1}^{n}l\left(y_{i},\hat{y_{i}}\right)+\sum_{k}\Omega \left(f_{k}\right), \end{array}$$

*l* represents the loss function between the observed value $y_{i}$ and the predicted value ${\hat{y}}_{i}$. In addition,

$$\Omega \left(f\right)=\gamma T+\frac{1}{2}\lambda {∥w∥}^{2}.$$

Ω is the regularized term that penalizes the complexity of the model to avoid overfitting of the data, γ penalizes the number of leaves or equivalently the complexity of the tree, *T* is the number of leaves in the tree, λ is the regularization parameter, and *w* is the score vector in the leaves.

The loss function is considered as the mean square error if the problem is regression, whereas in a binary classification problem, the loss function is the likelihood function obtained from Equation (2):

Let ${\hat{y}}_{i}^{\left(t\right)}=\sum_{k=1}^{t}f_{k}\left(x_{i}\right)=\left\{\sum_{k=1}^{t-1}f_{k}\left(x_{i}\right)\right\}+f_{t}\left(x_{i}\right)$, and Equation (4) can be written as:

(5) $$\begin{array}{c} L^{\left(t\right)}=\sum_{i=1}^{n}l\left(y_{i},{\hat{y}}_{i}^{\left(t-1\right)}+f_{t}\left(x_{i}\right)\right)+\Omega \left(f_{t}\right). \end{array}$$

Using the second order Taylor expansion of the loss function and denoting by $I_{j}=\left\{i|q\left(x_{i}\right)=j\right\}$ for some fixed structure q(x) to the index set whose examples correspond to that leaf, we can rewrite (5) as:

(6) $$\begin{array}{ccc} L^{\left(t\right)} & = & \sum_{j=1}^{T}\left[\left(\sum_{i\in I_{j}}g_{i}\right)w_{j}+\frac{1}{2}\left(\sum_{i\in I_{j}}h_{i}+\lambda \right)w_{j}^{2}\right]+\gamma T. \end{array}$$

The internal sums of the Equation (6) over the index set $I_{j}$ of the leaf *j* is due to the fact that the examples present in that leaf have the same score. Subsequently, to find the optimal value of the parameters $w_{j}$ in each of the leaves, fixing q(x) and deriving Equation (6), it was determined that such a value is:

(7) $$w_{j}^{*}=-\frac{\sum_{i\in I_{j}}g_{i}}{\sum_{i\in I_{j}}h_{i}+\lambda }.$$

Substituting (7) in (6), we found that the minimum value is reached in:

$$L^{\left(t\right)}\left(q\right)=-\frac{1}{2}\sum_{j=1}^{T}\frac{{\left(\sum_{i\in I_{j}}g_{i}\right)}^{2}}{\sum_{i\in I_{j}}h_{i}+\lambda }+\gamma T.$$

In practice, the goal is to optimize one level of the tree at a time, so that branches are added iteratively to a tree. Thus, if a leaf is divided into two leaves and the index set of the examples that are on the left leaf and on the right leaf are $I_{I}$ and $I_{D}$, respectively, then the reduction in loss after division is given by:

(8) $$L_{split}=\frac{1}{2}\left[\frac{{\left(\sum_{i\in I_{I}}g_{i}\right)}^{2}}{\sum_{i\in I_{I}}h_{i}+\lambda }+\frac{{\left(\sum_{i\in I_{D}}g_{i}\right)}^{2}}{\sum_{i\in I_{D}}h_{i}+\lambda }-\frac{{\left(\sum_{i\in I_{I}\cup I_{D}}g_{i}\right)}^{2}}{\sum_{i\in I_{I}\cup I_{D}}h_{i}+\lambda }\right]-\gamma .$$

This last expression consists of the scores of the original leaf, the left leaf, the right leaf, and the regularization term. In addition, this formula is the gain in the loss reduction equation; therefore, it is used to evaluate the division with the candidates.

## 3. Results

### 3.1. Spatiotemporal Distribution of Daily Cases

**Figure 1:**
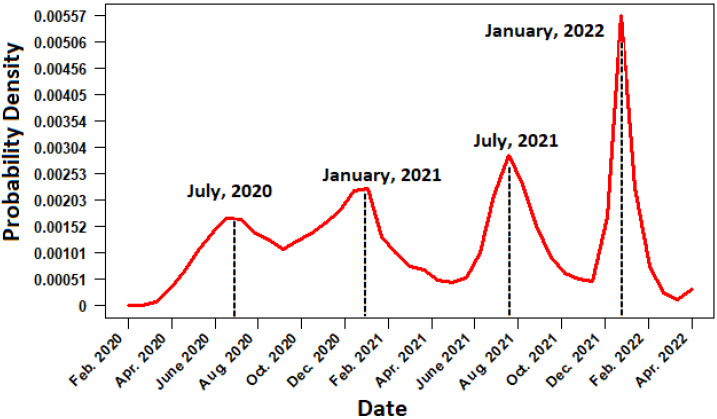
Temporal distribution function in terms of probability for daily COVID-19 cases from February 2022 to April 2021. The dotted line represents the date when a maximum was reached in COVID cases.

**Figure 2:**
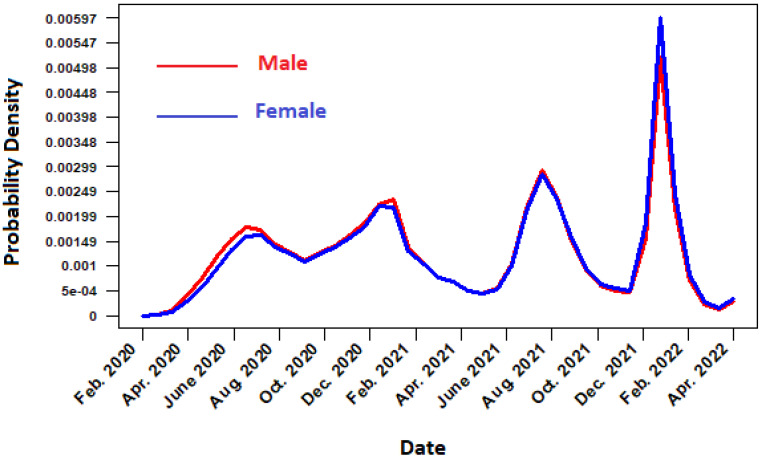
Temporal distribution function in terms of probability for daily COVID-19 cases by gender.

**Figure 3:**
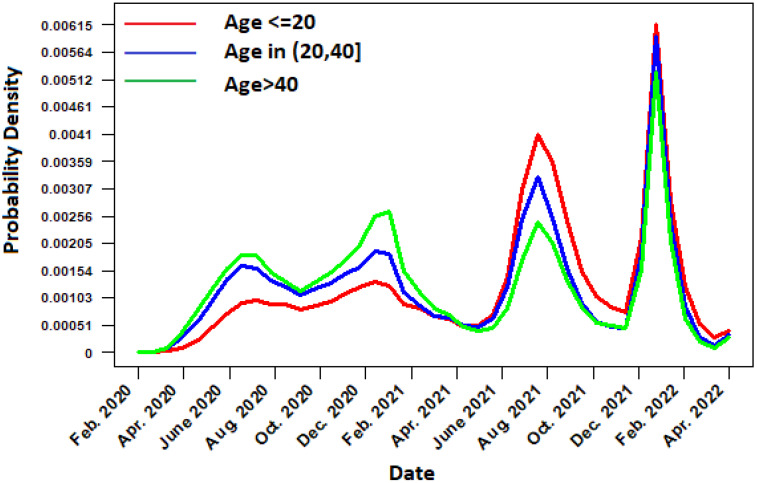
Temporal distribution function in terms of probability for daily COVID-19 cases by age.

The temporal distribution function of daily cases of COVID-19 in Mexico from February 2020 to the end of April 2022 is described in Figure 1. When analyzing the Figure 1, it can be seen that, after the arrival of the SARS-CoV-2 virus in Mexico, the daily cases of infected increased rapidly beginning in the month of May, reaching the peak of the first wave of positive cases in summer 2020 (mid-July and early August). In this first wave, the temporal distribution function considering the gender of the patient indicates that there is a greater presence of masculine cases than feminine. Subsequently, the second wave of positive cases is confirmed during the winter of 2021 (January–February). In this phase, the occurrence of positive cases was higher than in the first wave, the infection had propagated to every state in Mexico. In addition, notice from Figure 2 that the cases in women and men are quite similar, and the trend of male cases is very similar to the trend of female cases. On the other hand, the third and fourth wave of cases occurred during the months of July 2021 and January 2022. Furthermore, the last wave was the largest, with a significantly greater number of cases in men than in women. The distribution function by age group shows that, during the first and second wave, young people aged ≤20 were the least affected by the pandemic, while the most affected were people between 20 and 40 years, followed by adults older than 40 years. In the case of the third and fourth wave, there was an increased risk for young individuals (under 20 years), while older adults over 40 years decreased their chances of infection; see Figure 3.

**Figure 4:**
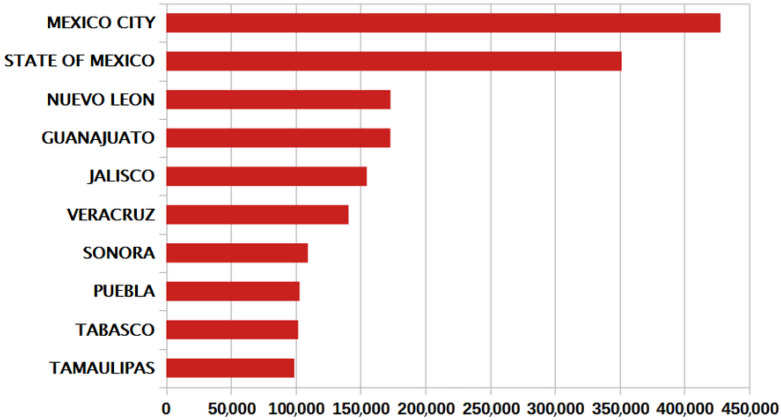
Mexican states with most COVID-19 cases from February 2020 to April 2022.

**Figure 5:**
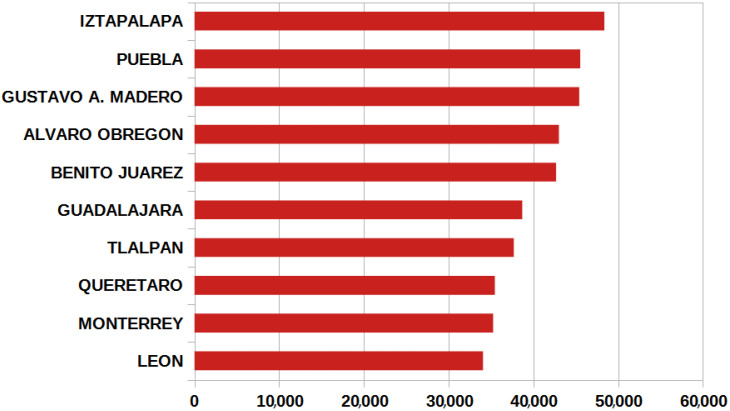
Municipalities with most COVID-19 cases from February 2020 to April 2022.

The results obtained from the statistical analysis by geographic location determined that the states with the highest occurrence of COVID-19 cases were the following: in the central zone of Mexico, the predominant states were Mexico City and the State of Mexico; in the west, Jalisco and Guanajuato; in the north of Mexico, these were Tamaulipas, Nuevo León, Sonora, and, in the south, these were Puebla, Veracruz, and Tabasco, see Figure 4. Most of the states least affected by the disease, with the exception of Chiapas, were the least populated, as is the case of Colima, Nayarit and Campeche; an exception is the state of Chiapas. On the other hand, the municipalities in Mexico with the most cases of COVID-19 were Puebla, Querétaro, León, Monterrey and Guadalajara. In the case of Mexico City, the largest number of cases occurred in the most populated municipalities, such as Iztapalapa, Gustavo A. Madero, Álvaro Obregón, Tlalpan and Benito Juarez, see Figure 5.

**Figure 6:**
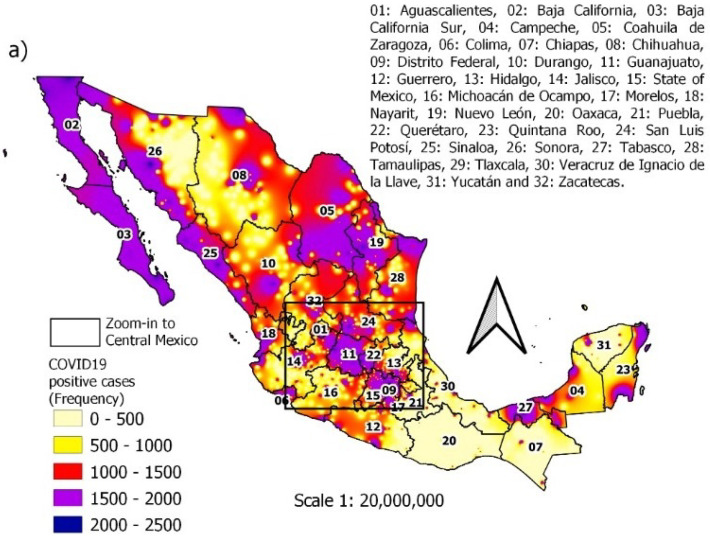

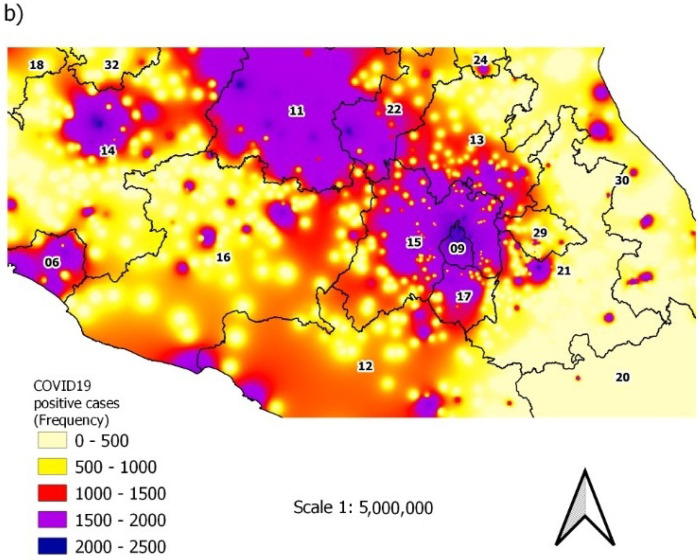
Spatial distribution of COVID-19 positive cases: (**a**) national context; (**b**) zoom-in to Central Mexico.

Additionally, the maps in Figure 6a,b show the actual frequency for each municipality of positive cases of COVID-19. Figure 6a shows that many entities in Mexico have a high number of incidents, while Figure 6b shows the center of Mexico, it can be seen that the federal district is one of the most affected entities.

### 3.2. Spatiotemporal Distribution of Individuals with Comorbidities

**Figure 7:**
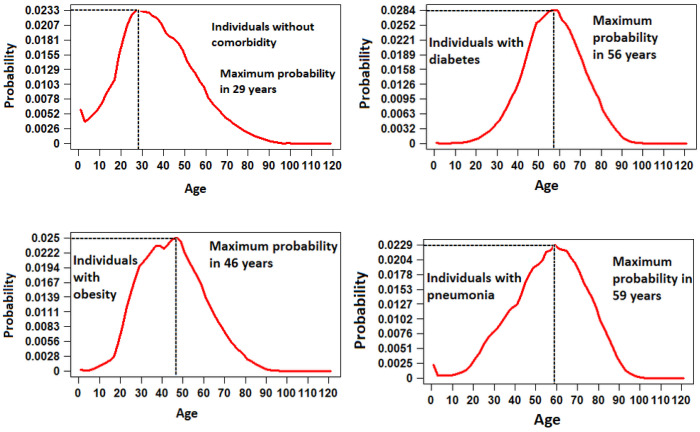
Probability distribution functions for positive COVID-19 cases by comorbidity against age.

In Mexico, a large part of the population suffers from a disease that exposes them to a higher risk of developing COVID-19 or of having serious complications once they are sick. Therefore, it is important to explore the probability of cases against the main risk factors. To do this, four groups that were diagnosed positive for COVID-19 were analyzed: (1) individuals who did not present any risk factor, (2) individuals with diabetes, (3) individuals who suffered from obesity and (4) those who presented pneumonia, Figure 7. For the group of individuals without comorbidity, it was determined that the highest number of incidences occurred in patients between the ages of 25 and 35 years, with an average of 37.75 years. In the case of individuals with diabetes, the mean was presented at 57.5 years, while, for obesity, the cases were positively skewed with a mean of 45.62 years, unlike pneumonia, where the distribution of cases was negatively skewed with a mean of 56.8 years.

**Figure 8:**
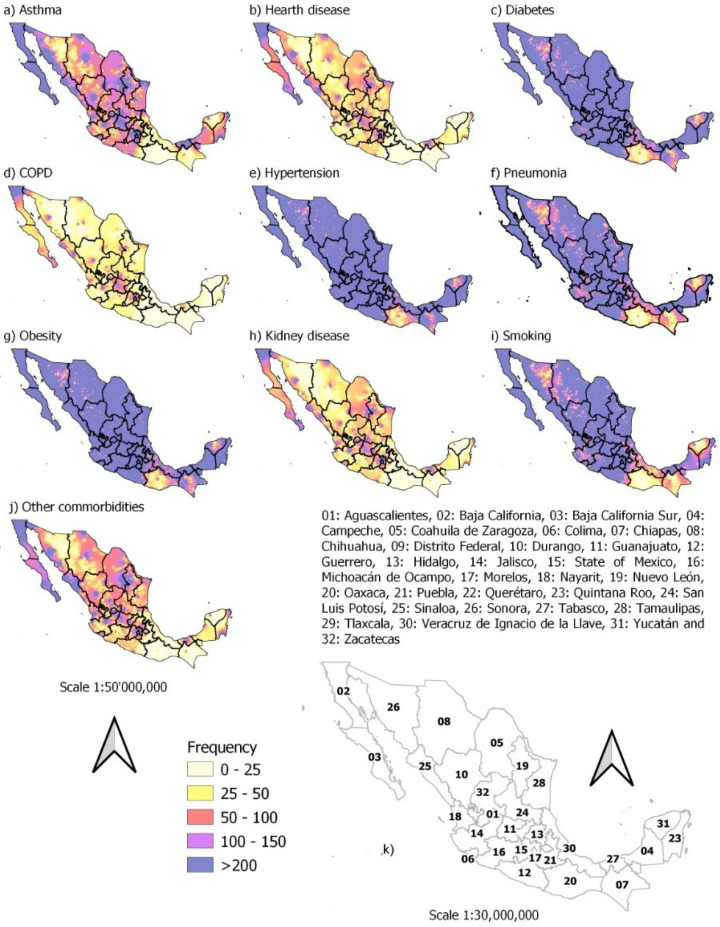
Spatial distribution of comorbidities associated with COVID-19 occurrence from February 2020 to April 2022: (**a**–**j**) all the comorbidities and (**k**) captions for states and countries.

The proportion of patients who presented some risk factor of the total set of records is described in Table 2. Figure 8 shows that the main risk factors present in a large part of the Mexican population are hypertension, pneumonia, obesity, diabetes and smoking. On the other hand, the spatial distribution of each of the comorbidities under study associated with the cases that tested positive for COVID-19 can be seen in Figure 8.

Table 3 shows the patients diagnosed as positive for COVID-19, who had some previous comorbidity. The main risk factors in the Mexican population included hypertension, pneumonia, obesity, and diabetes. In addition, only of the positive cases (3,119,851), 55.59% of the patients suffered at least one of the 10 diseases that were considered in the study. When analyzing the gender, it was found that 50.31% and 49.69% of the total positive cases are men and women, respectively. In the group of men, 56.16% had at least one disease, while in the case of women this was 55%.

### 3.3. Classification Analysis with XGBoost-LR and Classic LR

This study is focused on determining a model capable of predicting whether or not the patient will suffer from the COVID-19 disease, considering a set of explanatory variables. Additionally, it sought to identify the importance of each of these variables in different groups of patients. The study was performed with the XBGoost-LR model described in Section 2.3 using the likelihood of a multivariate logistic regression model as a loss function. The measure of selection that determined the order of importance of variables in the context of decision trees was the gain, defined in (8). This measure allowed for deciding which attribute should go to a decision node, selecting the one whose gain is greater.

**Figure 9:**
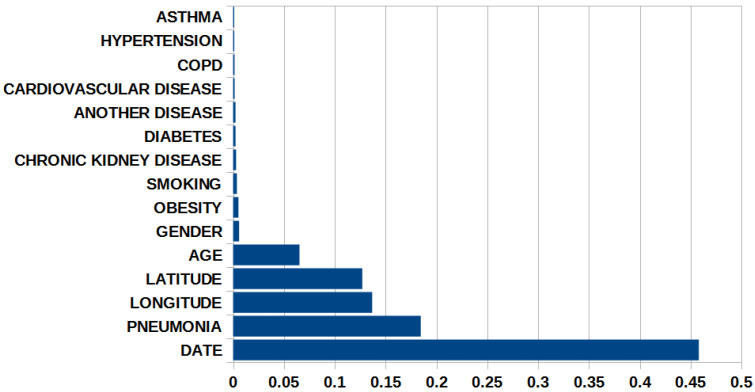
Importance of explanatory variables in XGBoost.

The XGBoost-LR model was built considering 70% of the data as the training set and 30% as the testing set. To measure model quality among a set of candidate models, evaluation metrics were calculated with only the test set. For the final model, we obtained a score known as sensitivity (66.11%) (i.e., recall rate or fraction of true positives), which is the proportion of positive cases that the model correctly identified. The specificity was 70.11% (true negative rate), which represented the proportion of negative cases that were correctly classified by the algorithm. The probability of how close the result of a measurement is to the true value, that is, the accuracy of the model, was 68.5%. In addition, it was determined that the most influential variables in the model (in terms of decision trees, which generate the greatest gain) for the classification were: date, pneumonia, location with latitude, longitude and age, while diseases such as asthma, hypertension, disease Chronic obstructive pulmonary disease and cardiovascular diseases were the least influential in the model classification. The importance of the variables is shown in Figure 9. This type of importance represents the average gain across all splits where feature was used. Therefore, the table shows that the variables age, latitude, longitude, pneumonia and date are the variables with the greatest importance, while that the variables associated with asthma, hypertension, COPD, cardiovascular disease, another disease, diabetes, chronic, kidney, disease, smoking obesity and gender have lower values of importance. Although the measure of importance obtained by these variables is low, this does not mean that the variables do not contribute to the adjustment; rather, it means that the number of cases in which the variable was decisive to form a new branch is lower.

The predictive capacity of the built model was analyzed by comparing it with other classification models. To do this, it was compared with the classic multivariate logistic regression model, which allows for predicting a binary response variable [23]. For training the classical logistic regression model, the same training and testing set used with the XGBoost-LR model was employed. The results obtained for the new model were as follows: the sensitivity was 43.63%, the specificity was 70.49%, and the accuracy was 59.69%. Therefore, it can be showed that the XGBoost-LR model outperformed the classic logistic regression model in almost all the assessment metrics, except for specificity, see Table 4.

**Figure 10:**
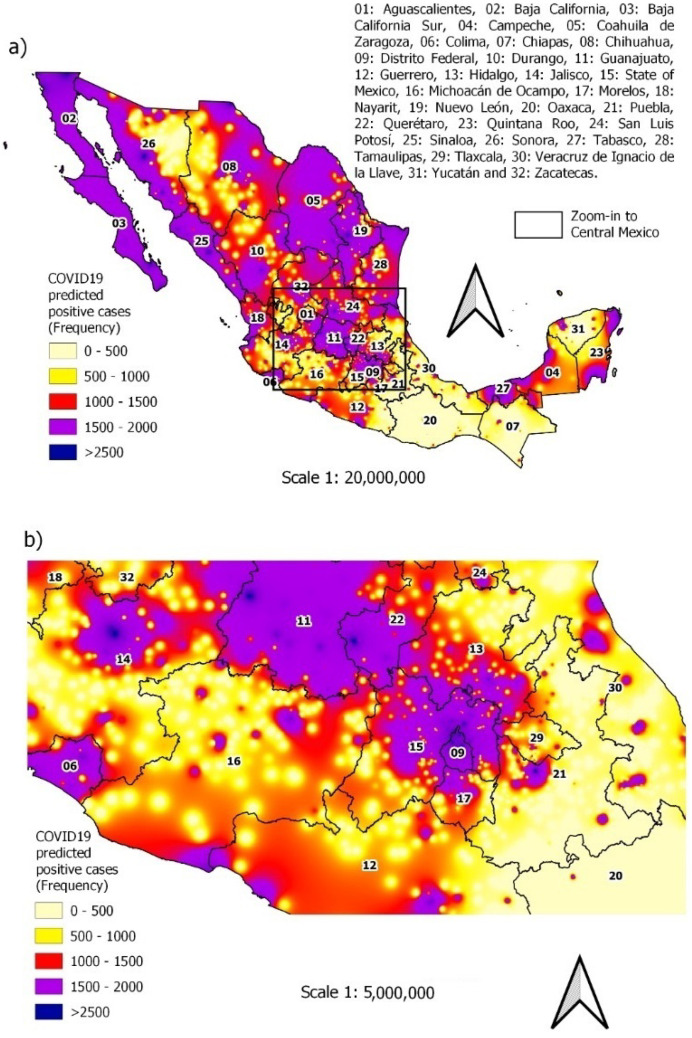
Predicted spatial distribution of COVID-19 occurrence from February 2020 to April 2022: (**a**) average actual occurrence with XGBoost-based logistic regression; (**b**) zoom-in to Central México.

**Figure 11:**
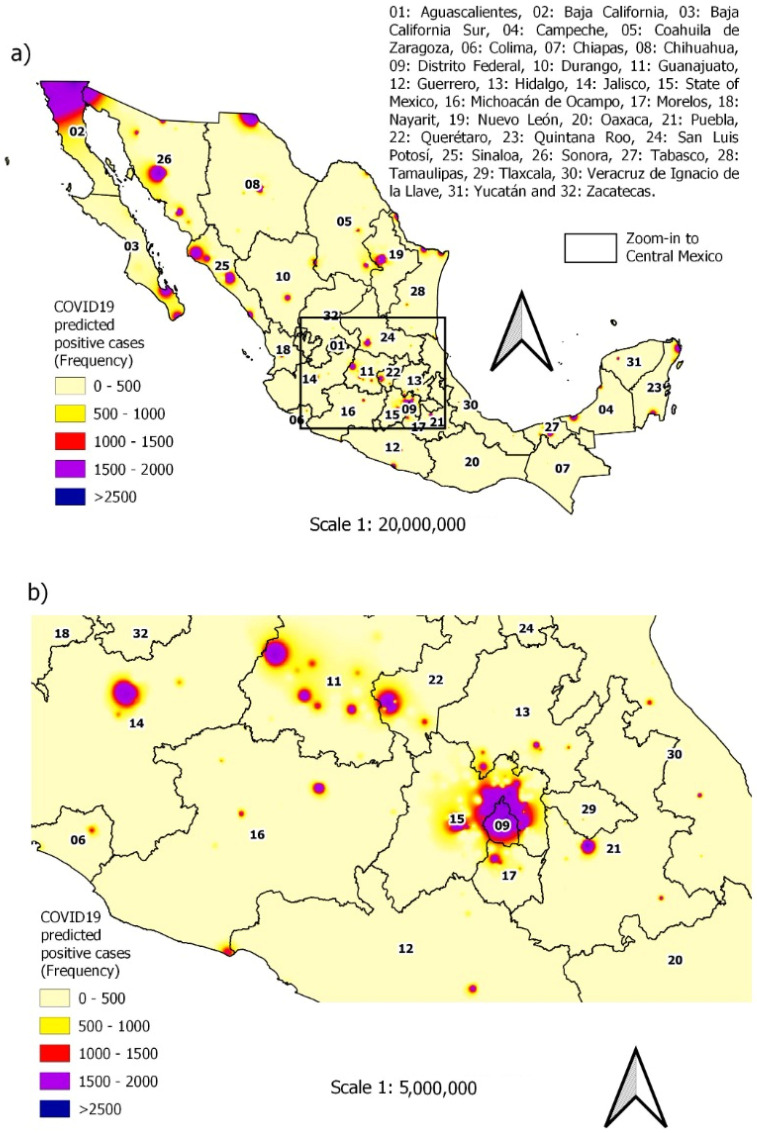
Predicted spatial distribution of COVID-19 occurrence from February 2020 to April 2022: (**a**) average actual occurrence with multivariate logistic regression; (**b**) zoom-in to Central México.

Alternatively, the predictive ability of the final model can also be viewed graphically using maps; see Figure 10a,b. The prediction of cases by municipality obtained with the XGBoost-LR model considering the entire Mexican territory can be seen in Figure 10a, while the zoom-in to Central Mexico is found in Figure 10b. Figure 10a shows the level of risk present in each of the municipalities, even more so the areas with the highest probability of risk include the north, coast and center of the country. Note that the map is quite similar to the map in Figure 6, so it can be assumed that the model has good predictive capacity on the data. We also elaborate a map with the predictions using the multivariate logistic regression model to contrast the results of the proposed model. The results are shown in Figure 11, in which we observe a significant difference with respect to the map of the observed values shown in Figure 6.

**Figure 12:**
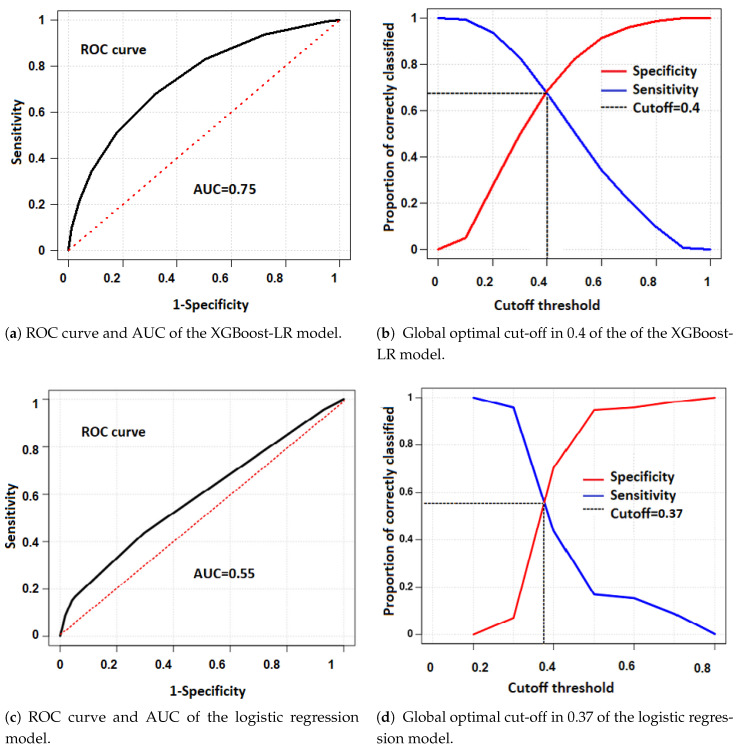
ROC curve (**a**,**c**), area under the AUC curve (**b**,**d**) and optimal cut-off value of the models: XGBoost-LR and logistic regression, respectively, to estimate the risk of suffering from COVID-19.

The XGBoost-LR model was analytically evaluated by calculating the area under the ROC curve called AUC. The AUC value obtained was 0.75, which indicates that, if an individual is randomly selected, then there is a probability of 0.75 that the diagnosis made by the model is correct. Thus, the constructed model is considered appropriate to predict the risk of acquiring the COVID-19 disease. Figure 12a, shows the ROC curve with the performance of the XGBoost model. Specificity evaluates the proportion of real negatives that are correctly identified, while sensitivity describes the proportion of real positives that were correctly identified. In this way, the ROC curve also provides a perspective on the behavior of the predictive potential of the proposed model. In addition, the optimal cut-off point was also determined, which maximizes the difference between sensitivity and 1-specificity (Figure 12b). Figure 12c shows the ROC curve and AUC obtained for the multivariate logistic regression model. The value of the AUC obtained for this model shows that the proposed model is better than the multivariate logistic regression model. In this model, the cut-off was 0.37, which is slightly lower than the cut-off obtained by the XGBoost model.

### 3.4. Predicting New Cases

Figure 9 shows that pneumonia is the most important variable of the variables associated with previous comorbidities. In addition, the rest of the variables associated with previous comorbidities also contribute to the construction of the model. The reason is because the decision tree considers spatial and temporal variables, and consequently, in certain geographical places, these variables play an important role for prediction. However, because these places are less numerous within the study region, the importance assigned to the variable is considerably lower. In the case of patients with no history, it was observed that the estimated probability of risk is low, so the aforementioned probability does not exceed the optimal threshold of 0.4, for which the model classifies these observations as negative cases for COVID-19. In the case of patients suffering from obesity and diabetes, it was determined that the probability of risk increases slightly compared to individuals who do not present comorbidity; however, this probability is also below the optimal threshold; thus, they are considered as cases negative for the disease. Finally, in the case of pneumonia, the probability of risk increases beyond the optimal threshold after 10 years.

## 4. Discussion

Our findings based on descriptive statistics determined that the most prevalent comorbidities in the studied population were obesity, diabetes, hypertension and pneumonia. When analyzed variables of location, the data reflected a notable spatial differentiation in the number of incidents of positive and non-positive COVID-19 cases. That is, urban areas in the central, northern and coastal region of the country, presented a high risk of infection compared to less populated places, such as Chiapas, Campeche and Colima, as can be seen in the maps.

Another result of the descriptive analysis was the ability to determine the time periods with a high rate of contagion in Mexico, which occurred shortly after the holiday seasons of each year. This is reasonable given the enormous mobility on those dates. Additionally, when analyzing the distribution function of positive cases of COVID-19 grouped by comorbidity, it was observed that older adults were more likely at least one of the comorbidities considered in the study. Depending on gender, there was almost the same proportion of positive cases in men and women, which suggests that the risk is independent of gender. On the other hand, after using the XGBoost-LR model, we were able to confirm that, indeed, a large part of the Mexican population was affected by the COVID-19 disease.

Our results also revealed that some vulnerable groups have a greater risk, which may be characterized by their health status and geographic location. This fact is of vital importance not only for the study of the new coronavirus disease, but to show the health status of a large part of the Mexican population. In fact, the Mexican Ministry of Health indicated that, after the United States, Mexico ranks second in terms of obesity rate, a disease that is highly related to diabetes, being one of the main causes of death in the country. On the other hand, the analysis of the geographical locations makes it possible to analyze the existing risk in densely populated places, and encourages the relevant authorities to take better measures, for example, to strengthen security protocols, in touristic areas of the country.

Treviño [24] found the presence of the same comorbidities, finding that men suffer from COVID-19 disease more often than women. In addition, he indicated that admission to the Intensive Care Unit was related to the gender patients, as verified by the Chi-square test. Our result probably differs in that, during the first year of the pandemic, there were more positive cases in men than in women; however, during the second year, this could change. On the other hand Ref. [15] performed a logistic regression analysis and determined that men were 1.54 times more likely to be hospitalized than women. Furthermore, both [25] and [9] found that, in addition to diabetes and obesity, high blood pressure and chronic kidney damage can increase mortality in COVID-19 patients. Regarding the analysis by geographic location of each state of Mexico, Refs. [2,26] analyzed the cases of COVID-19 in Mexico. In both studies, the results agreed with our analysis, since the same spatial pattern was detected throughout the country: State of Mexico, Nuevo León, among others, were the most affected by the pandemic.

Having a predictive model that allows for identifying individuals with particular characteristics is important for taking preventive measures to avoid further deaths. On the other hand, the constant appearance of new variants of the virus worries stakeholders around the world and makes it difficult to pinpoint an end date for the pandemic. Therefore, the authorities must strengthen surveillance and adopt systematic approaches to provide health indications to the entire population, based on each local context.

## 5. Conclusions

In the present study, positive and non-positive cases of COVID-19 in Mexico were analyzed at the municipal level using data from the Mexican Ministry of Health. A descriptive analysis was performed to explore the spatial and temporal distribution of positive cases, and subsequently an XGBoost-LR model was trained to estimate the risk of infection using the main comorbidities in patients as covariates. According to the results, we confirm that pneumonia, obesity and diabetes were variables of utmost importance to predict new cases. The estimated and observed values of percentage occurrence of cases were very similar, and indicated that the proposed model was suitable to predict new cases (AUC = 0.75). The optimal global cutoff to identify when a case is positive for COVID-19 was 0.41. Our results revealed that patients without comorbidities are less likely to be COVID-19 positive, unlike people with diabetes, obesity and pneumonia with ages close to 56, 46 and 59 years old, respectively. The current worldwide situation regarding COVID-19 is still serious, especially with the emergence of new SARS-CoV-2 virus variants. Therefore, we recommend reviewing and adapting government policies concerning the establishment of preventative measures to avoid the spread of virus and mitigate the pandemic effects.

## Figures and Tables

**Table 1:** Explanatory variables used in the study.

	Variable	Description	Units	Input Data Source ^1,2^
1	Date	−	Days	DGE
2	Gender	0: Male, 1: Female	−	DGE
3	Age	−	Years	GDE
4	Asthma	0: No, 1: Yes	−	DGE
5	Diabetes	0: No, 1: Yes	−	DGE
6	Cardiovascular disease	0: No, 1: Yes	−	DGE
7	COPD	0: No, 1: Yes	−	DGE
8	Hypertension	0: No, 1: Yes	−	DGE
9	Obesity	0: No, 1: Yes	−	DGE
10	Chronic kidney disease	0: No, 1: Yes	−	DGE
11	Smoking	0: No, 1: Yes	−	DGE
12	Pneumonia	0: No, 1: Yes	−	DGE
13	Other disease	0: No, 1: Yes	−	DGE
14	Longitude	X UTM coordinate	m	CONABIO
15	Latitude	Y UTM coordinate	m	CONABIO

^1^https://www.gob.mx/salud/documentos/datos-abiertos-152127 accessed on 10 May 2022; ^2^ http://www.conabio.gob.mx/informacion/gis/maps/geo/cabmun2kgw.zip accessed on 10 May 2022.

**Table 2:** Percentage of patients with any comorbidity, out of a total of 7,771,714 observations.

Variable	Yes	No
Hypertension	19.00%	81.00%
Pneumonia	9.00%	91.00%
Obesity	15.50%	84.50%
Diabetes	14.17%	85.83%
Smoking	10.02%	89.98%
Asthma	3.77%	96.23%
Chronic kidney failure	1.88%	98.12%
Cardiovascular disease	1.94%	98.06%
Other disease	2.6%	97.40%
COPD	1.32%	98.68%

**Table 3:** Percentage of COVID-19 patients with any comorbidity, out of a total of 3,119,851 observations.

Variable	Yes	No
Hypertension	21.62%	78.38%
Pneumonia	15.62%	84.38%
Obesity	17.67%	82.33%
Diabetes	16.53%	83.47%
Smoking	8.98%	91.02%
Asthma	3.37%	96.63%
Chronic kidney failure	1.93%	98.07%
Cardiovascular disease	1.95%	98.05%
Other disease	2.78%	97.22%
COPD	1.37%	98.63%

**Table 4:** Evaluation metrics of the XGBoost-LR and classic multivariate logistic regression models.

Model	Sensitivity	Specificity	Accuracy
XGBoost	66.11%	70.11%	68.50%
Multivariate logistic regression	43.63%	70.49%	59.69%

## Data Availability

The analyzed data sets can be consulted at the following links: https://www.gob.mx/salud/documentos/datos-abiertos-152127 accessed on 10 May 2022; http://www.conabio.gob.mx/informacion/gis/maps/geo/cabmun2kgw.zip accessed on 10 May 2022.

## Abbreviations

The following abbreviations are used in this manuscript:

COVID-19	`CO’ stands for corona, ‘VI’ for virus, ‘D’ for disease, and ‘19’ for 2019
CONABIO	National Commission for the Knowledge and Use of Biodiversity
COPD	Chronic obstructive pulmonary disease
DGE	Directorate General for Epidemiology
ROC	Receiver Operating Characteristic
SARS-CoV-2	Severe acute respiratory syndrome coronavirus 2
WHO	World Health Organization
